# Assessment of cognitive biases in Augmented Reality: Beyond eye tracking

**DOI:** 10.16910/jemr.15.3.4

**Published:** 2022-12-30

**Authors:** Piotr Słowiński, Ben Grindley, Helen Muncie, David J Harris, Samuel J Vine, Mark R Wilson

**Affiliations:** University of Exeter, United Kingdom; Defence Science and Technology Laboratory, United Kingdom; Thales, United Kingdom

**Keywords:** Eye movement, hand movement, head movement, earth mover’s distance, correlation matrix, eye tracking, cognitive bias, augmented reality

## Abstract

We study an individual’s propensity for rational thinking; the avoidance of cognitive biases (unconscious errors generated by our mental simplification methods) using a novel augmented reality (AR) platform. Specifically, we developed an odd-one-out (OOO) game-like task in AR designed to try to induce and assess confirmatory biases. Forty students completed the AR task in the laboratory, and the short form of the comprehensive assessment of rational thinking (CART) online via the Qualtrics platform. We demonstrate that behavioural markers (based on eye, hand and head movements) can be associated (linear regression) with the short CART score – more rational thinkers have slower head and hand movements and faster gaze movements in the second more ambiguous round of the OOO task. Furthermore, short CART scores can be associated with the change in behaviour between two rounds of the OOO task (one less and one more ambiguous) – hand-eye-head coordination patterns of the more rational thinkers are more consistent in the two rounds. Overall, we demonstrate the benefits of augmenting eye-tracking recordings with additional data modalities when trying to understand complicated behaviours.

## Introduction

Neoclassical or rational models of decision-making suggest that humans make decisions based on full and relevant information (13). However, as Herbert Simon (43, p. 500) stressed, the classical model of rationality requires, “knowledge of all the relevant alternatives, their consequences and probabilities, and a predictable world without surprises”. Many professionals on the other hand, face an operating environment characterized by volatility, uncertainty, complexity, and ambiguity (56). In such environments, intuitive decision making often necessitates the use of mental heuristics – or ‘rules of thumb’ - to quickly reduce complexity. The price to pay for the speed and efficiency associated with heuristics, is that they require generalization and the neglect of some potentially important information. As such, heuristics allow people to make “good enough” choices - a trade-off between effort and potential accuracy - in a sensory-rich and complex environment (10; 37).

Four interesting issues become pertinent when considering how heuristics might be used to guide decision-making in complex environments: (1) How might cognitive biases influence the information that is selected for processing; (2) How might we objectively detect how this information is attended to and processed; (3) Can we categorise individuals based on information selection tendencies; and (4) Can we provide support to overcome potential errors in decision-making due to biases. The aim of this study was to initiate enquiry into the first three of these issues and specifically, to show potential for quantifying an individual’s propensity for rational thinking using a novel augmented reality platform. Existing tools for assessment of rational thinking have form of rather lengthy and abstract questionnaires (48). Identifying objective markers of important elements of rational thinking that potentially could be measured in real-time would greatly expand this area of research (5).

While heuristics can be useful (see 13; 37), they can lead to the injection of cognitive bias - unconscious errors generated by our mental simplification methods (56). Discussion of heuristics and biases often leads to a conceptualization within a dual-process framework because most of the tasks in the heuristics and biases literature have been deliberately designed to pit an automatically triggered response (Type [System] 1) against a normative response generated by more controlled types of processing (Type [System] 2) (19). In these tasks, the subject must detect the inadequacy of the Type 1 response and then must use Type 2 processing to both suppress the Type 1 response and to simulate a better alternative (48).

The dominance of Type 1 versus Type 2 processing in determining a final decision, tends to be assessed via specific cognitive tasks (e.g., the Comprehensive Assessment of Rational Thinking, CART; (48)). However, there is increasing interest in capturing objective process measures of intuitive (non-rational) decision-making. One such process measure is eye movements, which provide “a window into our mind and a rich source of information on who we are, how we feel, and what we do” (16, p1). As such, eye movement metrics may provide insights as to how someone will make decisions under certain circumstances (see 32, for a review).

Most of the work examining eye movements as biometric markers has been directed to the early detection of neurological and clinical disorders such as autism (39) or Alzheimer disease (4). Recently, these applications have utilised novel mathematical and machine learning approaches. For example, Tseng et al. (54) used machine learning to identify critical features that differentiated patients from control subjects based on their eye movement data while watching 15 minutes of television. They classified Parkinson’s disease versus age-matched controls with 89.6 % accuracy (chance 63.2 %), and attention deficit hyperactivity disorder versus fetal alcohol spectrum disorder versus control children with 77.3 % accuracy (chance 40.4 %).

Importantly, it is not just brain dysfunction that may be detected via analyses of eye movements, but more subtle psychological differences. Optimists, for example, spend less time inspecting negative emotional stimuli than pessimists (17), and extraversion influences fixation time of people-based images (28), 2012). Individuals high in openness spend a longer time fixating and dwelling on locations when watching abstract animations (38), and perceptually curious individuals inspect more of the regions in a naturalistic scene (40). More recently, Hoppe et al. (16) tracked eye movements while participants ran an errand on a university campus. They revealed that the visual behaviour of individuals engaged in an everyday task can predict four of the Big Five personality traits (neuroticism, extraversion, agreeableness, and conscientiousness) as well as perceptual curiosity. Building on Hoppe’s work, Woods et al., (57) demonstrated that using just twenty seconds of visual behaviour on social-media gives insight into personality traits.

Our approach therefore extrapolates from two fields: recent psychometric work examining how task related eye movements can predict personality traits (e.g., 16) and our own work unravelling socio-motor biomarkers in schizophrenia through the identification of individual motor signatures (coordinated hand movements) (46, 44, 45). There is exciting potential to model eye *and* hand movements in the completion of goal-directed tasks that might act as biomarkers of the underlying cognitive processes that support the completion of these tasks. Recently, researchers have started to consider combining gaze behaviour with other movement modalities for identity classification (25; 35) as well as personality trait predictions (26), using extended reality and other experimental set-ups.

We therefore aim to quantify an individual’s propensity for cognitive biases using a novel augmented reality (AR) platform as the first step in developing a suite of tools to mitigate biases in operators of defence and security systems. We choose the AR platform, instead of a computer or a smartphone, to showcase and explore feasibility of collecting multimodal behavioural data in AR scenarios. The AR headsets have a large range of proposed applications, from construction and engineering, through healthcare to defense and security, which potentially could benefit from behavioural monitoring. Specifically, we aimed to demonstrate that we can (1) create tasks in augmented reality that might reflect elements of rational thinking, and (2) compute behavioural markers of performance for these tasks that correlate with psychometric measures of rational thinking.

As is typical of machine learning studies that seek to associate biological measures with psychometric ones (e.g., 16; 46), this work was primarily exploratory, therefore we had no a priori hypotheses other that such an association exists.

## Methods

### Participants

40 participants (age 18+ years) from the student population at the University of Exeter volunteered to take part in the study; data about age and gender of the participants was not collected in the Qualtrics platform (https://www.qualtrics.com). Participants who needed to wear corrective glasses (contact lenses were allowed) to use a computer were excluded from the study. The reason being that the eye-tracker would not fit together with the glasses under the headset. Participants did not have any experience in using the experimental set-up consisting of the AR headset with leap motion sensor. Participants were paid £10 for completing both elements of the study (the online psychometric tests and the augmented reality tasks). The online psychometric tests took about 2 hours and the laboratory session took about 30 minutes. Participants completed two tasks in AR; an odd-one-out task and a mirror game task (based on 46). They also completed the HEXACO-60 personality inventory (3). As we were interested in exploring rational thinking in this paper, we did not include data from either the mirror game task or the HEXACO-60 in our analyses. Ethical approval for the study was provided both by the University of Exeter (190506/A02) and MODREC (971/MoDREC/2022) and participants provided written informed consent before taking part.

### Design

This study adopted a correlational design, with validated psychometric measures of rational thinking correlated with performance and gaze/hand/head movement data in the augmented reality tasks.

### Materials

Psychometric Measure: Comprehensive Assessment of Rational Thinking (CART: 48). The CART is a comprehensive framework for measuring rational thinking and considers a range of thinking errors, related to both *miserly processing* - the tendency to use shortcuts and heuristics to make decisions when processing demands are high and, *mindware problems* - reflecting errors caused by missing (mindware gaps) or incorrect knowledge (contaminated mindware). Mindware is a label for the rules, knowledge, procedures, and strategies that a person can retrieve from memory to aid decision making and problem solving.

The full assessment takes about 3 hours to complete, while a short version takes ~2 hours (scored out of 100, with a higher score reflecting more rational thinking). We decided to use the short form in this project for expediency and because normative data does exist. The short-form has a Cronbach's alpha of 0.76 (calculated by treating subtests as items with no differential weighting of CART points allocated – 49). The CART was presented using the Qualtrics platform, which participants accessed via an email link. The Qualtrics items were provided by the lead author of the instrument once evidence that the accompanying book had been purchased was provided (48) and a research contract signed by the principal investigator (MRW).

**Figure 1.**
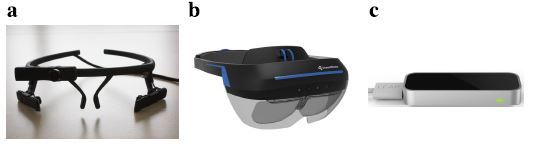
Hardware images. **a.** Pupil-labs eye-tracker. **b.** Dream Glass augmented reality goggles. **c.** Leap motion tracker.

Measures from the AR tasks were collected using the AR goggles’ sensors (head movements), a Leap motion tracker (for hand movements,) and an eye-tracker (eye movements) and subsequently modelled (see Figure 1 for hardware images and Table 1 for hardware specification).

**Table 1.** Hardware specifications

	Eye movements	Head movements	Hand movements
Equipment	Pupil labs (20)	DreamGlass Developer Edition 2019 (9)	Leap motion (31; 55)
Max capture rate	200 Hz	60 Hz	100 Hz
Placement	On head (under AR goggles)	On head	On table in front of a participant

Tasks were developed by a professional software developer with multiple rounds of feedback and revisions to decide size of images, spatial layout, and viewing distance. Feedback to developer was provided by mainly by PS and to lesser extend by BG, HM and MRW. Tasks were developed using the Unity Real-Time Development Platform (Unity 2019.2.17f1) and task specific functionality was coded in C# programming language. To interface with the equipment we used Dreamworlds glasses unity SDK (DreamWorld_2018.3.6.unitypackage), Leap Motion unity SDK (Unity Core Assets 4.4.0) and Pupil-labs unity SDK (Hmd-Eyes.VR.v1.1.unitypackage). Tasks used graphical assets obtained from the Unity Asset Store. The design did not control focal plane distance (potentially inducing vergence-accommodation conflict). Two tasks were originally designed; a mirror game task based on Slowinski et al.’s (46, 44) studies in patients with schizophrenia, and an odd-one-out task designed to test cognitive biases. In this paper we will only focus on the odd-one-out task.

### Procedure

**Figure 2.**
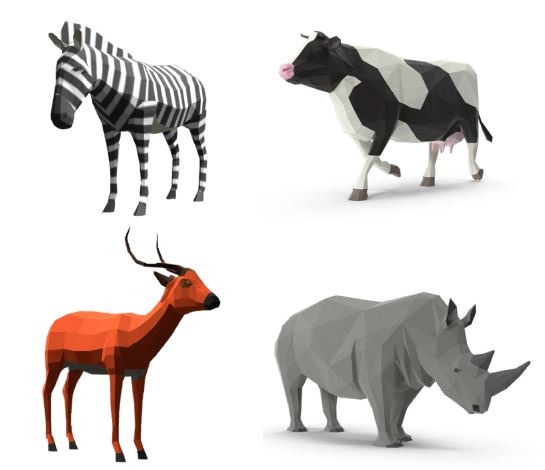
An example of the ambiguous odd-one-out task – zebra has no horns, rhino has no fur, and cow is a domesticated animal. Graphics are assets from the unity asset store.

The odd-one-out task used in the study is based on similar tasks that are used to measure deductive reasoning abilities (41). The task involved looking at four objects or animals displayed on 2-by-2 grid and deciding which is the odd-one-out based on a number of factors (e.g., colour, shape, ‘natural’ environment - see Figure 2). The task is designed to be ambiguous without one fully right answer. Once participants decided on an option, they reached out to ‘touch’ the displayed object to confirm their choice. Participants were allowed to use either hand to ‘touch’ the displayed object.

Participants completed two rounds of the task, which are referred to as OOO_1_ and OOO_2_. The first round (OOO_1_; 12 trials) is relatively simple, with mostly inanimate objects (e.g., chairs, tables, cars). The instruction given to the participant before the 1st round is: ‘You will now play an odd-one-out game. You will see a series of 12 sets of 4 objects. Please use your hand to select the object that is different, the “odd one”. There are multiple reasons why each object could be the odd one out. In half of the trials, selected at random, you will be presented with a possibility to change your selection.’

In the second round (OOO_2_; 13 trials) the possible options are more ambiguous, with at least two choices between animals that could be picked out as the odd one (see Figure 2 for an example). The full instructions read: ‘You will now play the odd-one-out game again. This time the game will be more ambiguous than previously. Instead of objects you will see 13 sets of 4 animals. There are multiple reasons why each animal could be the odd one out (e.g. the place where it lives). Again, in half of the trials, selected at random, you will be presented with a possibility to change your selection.’.

The opportunity to change their initial response was provided to participants in half of the trials in each round (randomly allocated with probability 1/2). At this point, participants were also presented with additional information aimed to challenge their initial response. For example: ‘Have you noticed that: only the rhino has no fur, only the zebra has no horns, only the cow is a domesticated animal. Please make a new selection or make the same selection again.’ As such, the odd-one-out task aimed to quantify elements of confirmation bias during visual inspection tasks (e.g., 30) - the tendency to look for evidence that confirms our initial beliefs. The sequence of OOO_1_ and OOO_2_ (including the order of individual trials) was the same for all participants. A list with the description of all the 25 sets of OOO tasks and the additional information messages to challenge the initial response can be accessed at osf.io/kcd83.

Participant’s choices, as well as head, hand and eye movement data were recorded for subsequent analysis. In the analysis, we compare the data collected in the two rounds.

Task measures computed for each round are: ratio of changed decisions – normalised number of times a person changed decision after the initial response (1^st^ decision) if presented with opportunity to do so; mean time of the 1^st^ decision; mean time of the 2^nd^ decision; total time – time from presentation of the 1^st^ trial to the last decision in the last trials of the round.

### Data collection and pre-processing

We excluded 4 participants that did not complete the questionnaires. We further excluded 4 participants that completed the questionnaires in under 62 minutes as it was felt that their responses were likely to be insufficiently thought out. We based the cut-off value on the fact that nearly all respondents typically complete the HEXACO-60 in 12 minutes (15) and the assumption that it takes at least 50 minutes to complete the short CART battery of questions (majority of respondents completed it in under 75 minutes, and nearly all completed it under 100 minutes). We further excluded any participants that had less than 60 seconds of valid datapoints; separately for each data modality and across them for analysis of correlation patterns. See summary in Table 2.

**Table 2.** Datasets available after exclusions.

	CART	Head	Hand	Gaze	Corr.
OOO_1_	32	31	29	24	24
OOO_2_	32	32	31	26	25

Note. CART – CART scores, Head – head movements recordings, Hand – hand movements recordings, Gaze – gaze recordings, Corr. – correlation matrices (lower number of available correlation matrices is due to misalignment of intervals of missing data in different recordings) misalignment of intervals of missing data in different recordings).

Head rotations were recorded using dreamworld augmented reality goggles Developer Edition 2019 (9) and transformed from 0–360 degrees range to -180–180 degrees range and resampled at 10Hz.

Hand movements were recorded using leap motion sensor (31; 55). Before analysis they were resampled at 10 Hz and had first and last 2 seconds of data removed.

Eye-tracking data was recorded using pupil-labs trackers (20). Before analysis we: (1) removed any gaze data points with position estimation confidence below <0.6, (2) removed points with coordinates below 0 and above 1 (which indicate that gaze was pointing outside the world frame), and (3) resampled the data at 60Hz. To detect saccades in the gaze data we followed well-accepted conventions (11). Saccades were detected by searching for samples where velocity exceeded a.u./sec, peak acceleration exceeded 90 a.u./sec^2^ and total distance traveled during saccade exceeded 0.005 a.u. Here a.u. is a ratio of the normalized world frame (diagonal FOV of the DreamGlass is 90^o^, approx. 74 ^o^ horizontal and 51 ^o^ vertical).

For calibration we used the Pupil Labs calibration for head mounted displays (36) adapted to use with DreamGlass AR headset. We were unable to adjust the inter-pupillary distance of the DreamGlass Developer Edition 2019 headset, as the SDK used in the study did not provide this option.

**Figure 3.**
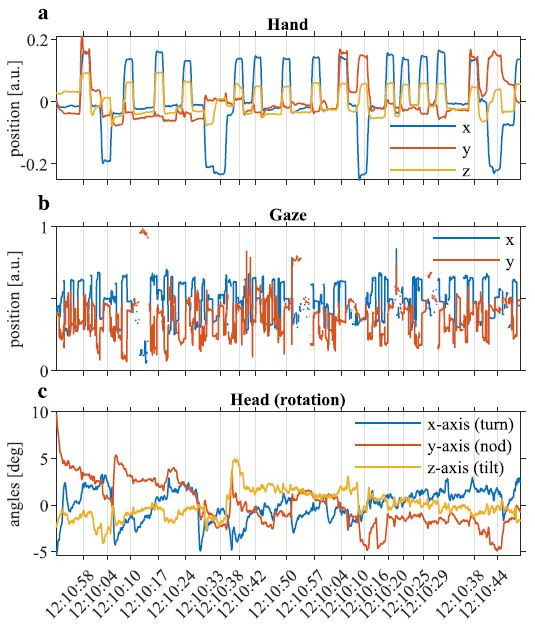
Visualisation of an example of the data collected in the odd-one-out task. **a.** hand, **b.** gaze and **c.** head time-series; vertical lines indicate times at which participant selected an object. In **a.** x (blue) corresponds to the left-right movement; y (orange) corresponds to up-down movement; z (yellow) is forward-backward motion. In **b.** x (blue) corresponds to the left-right movement; and y (orange) corresponds to up-down movement. In **c.** blue indicates left-right rotation along x-axis (turn); orange indicates up-down rotation along y-axis (nod); yellow indicates left-right rotation along z-axis (tilt). The visible discontinuities in the gaze data are due to missing data (confidence < 0.6).

An example of the aligned time series for one participant is shown in Figure 3.

### Biometric measures

To assess the associations between the recorded data and short CART score we first transformed the time-series into data representations that could be considered objective markers of rational thinking propensity. Following our earlier work (46, 44, 45), we used distributions and correlation matrices.

### Velocity distributions

**Figure 4.**
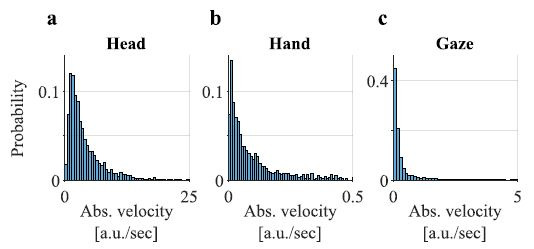
Examples of the histograms of the total velocity of **a.** head, **b.** hand and **c.** gaze. The ranges of the velocities are reduced in comparison with the ranges used for analysis.

Specifically, we analysed distributions of absolute total velocities, $v_{\text{tot}}=\sqrt[{2}]{v_{x}^{2}+v_{y}^{2}+v_{z}^{2}}$, of the head, hand and gaze (just 2 dimensions so: $v_{\text{tot}}=\sqrt[{2}]{v_{x}^{2}+v_{y}^{2}})$. In comparison with point measures (e.g., means, medians, standard deviations) distributions preserves significantly more information about a sample and thus allows for more accurate analysis. To approximate the distributions, we used histograms with bin edges obtained when applying the Freedman-Diaconis rule (12) to the combined velocity values from all the datasets of a given modality. The rule is particularly suitable for velocity data with heavy-tailed distributions (see examples in Figure 4).

### Earth mover’s distance

To quantify similarities between the distributions we used Earth mover’s distance (EMD). Intuitively, the EMD is the minimal cost of work required to transform one ‘pile of earth’ into another; here each ‘pile of earth’ represents a probability distribution. EMD has been widely used in computer and data sciences (24; 29). For univariate probability distributions, the EMD has the following closed form formula (7):

$$\text{EMD}\left(\text{PD}F_{1}\left(z\right),\;PDF_{2}\left(z\right)\right)=\int_{z}^{}\left|\text{CD}F_{1}\left(z\right)-CDF_{2}\left(z\right)\right|\text{dz}.$$

Here, $\text{PD}F_{1}$ and $\text{PD}F_{2}$ are probability density functions being compared, while $\text{CD}F_{1}$ and $\text{CD}F_{2}$, are their respective cumulative distribution functions. Z is the support set of the PDFs. To compute the EMD, we first find the experimental CDFs of the distributions of total velocities. We then interpolated the CDFs at the same points for each distribution.

### Correlation matrices

**Figure 5.**
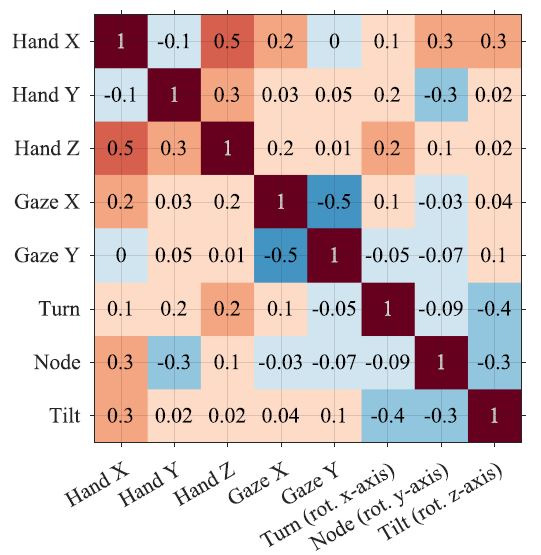
An example of a correlation matrix. Each entry in the correlation matrix is a Pearson’s correlation coefficient between two timeseries. The values are colour coded with negative values in blue and positive values in red; darker shades indicate lower/ higher values.

To obtain correlation matrices we computed all the pairwise Pearson’s correlation coefficients between the aligned time-series of hand (x, y and z coordinates), eyes (x and y coordinates) and head movements (rotations along x, y and z axis). In this way we obtained 8x8 symmetric matrices with all the values between -1 and 1 (see example in Figure 5).

### Riemannian distance

To quantify similarities between the coordination patterns (correlation matrices) we applied Riemannian geometry approach which is mathematically suitable for their analysis (8; 21). The Riemannian distance (RD) between two correlation matrices $C_{1}$ and $C_{2}\;$is given by:

$$\text{RD}\left(C_{1},C_{2}\right)=\sqrt{\sum_{n=1}^{N}\log^{2}\lambda_{n}}\;,$$

where $\lambda_{n}$ are the N eigenvalues of a matrix $C_{1}^{-1/2}C_{2}C_{1}^{-1/2}$ (or equivalently $C_{1}^{-1}C_{2}$) (8; 45).

### Data analysis

To quantify and assess existence of associations between short CART scores and our objective markers we computed correlations of the short CART scores with total velocity distributions or coordination patterns, and task outcomes, using two statistical methods: a combination of multi-dimensional scaling (MDS) with regression analysis and bias corrected distance correlation (BCDC) (53). To interpret the findings we additionally, computed correlations of the short CART scores with mean velocities of the head, hand and gaze movements and saccades rate (number of saccades per second). Description of the BCDC method is presented in Supplementary Note 1. Results of the analysis by means of BCDC are presented in the Supplementary Table 1. We used the BCDC to verify the findings using an alternative method. We include the BCDC analysis in the supplement for the sake of transparency and to show that our conclusions (in a broad sense) can be reached using different statistical methods.

### Multi-dimensional scaling

We further employed the multidimensional scaling space (MDS) to transform the similarity/ distance matrices into points in an abstract geometric space; MDS is similar to principal components analysis (PCA) in which similarity between variables is measured using correlation (see also 46, 45). In this abstract geometric space, each dataset is represented as a single point, and distances between the points are proportional to how similar they are, i.e., similar points are located closely together. Higher MDS dimensions represent smaller ratio of variability (like in the case of higher principal components).

### Regression analysis

The coordinates of the points computed by means of MDS allow an alternative way of quantifying and assessing existence of associations between the short CART scores and movements. To this end we employ linear regression models estimated using stepwise regression. After the initial fit, the stepwise regression examines a set of available terms, and adds the best one to the model if an F-test for adding the term has a p-value of 0.05 or less. If no terms can be added, it examines the terms currently in the model, and removes the worst one if an F-test for removing it has a p-value 0.10 or greater. It repeats this process until no terms can be added or removed. The function never removes the constant term. To avoid potential pitfalls of the stepwise regression (47) we only considered models that showed significant correlation with a single MDS coordinate and we only considered correlations with the first 10 dimensions.

Finally, we adopt robust regression, using the bi-square weighting function (50), to estimate the R^2^ coefficients and significance levels for any reported regression analysis. The advantage of the robust regression is that it reduces outlier effects in linear regression models. The p-value of the robust regression is based on the F-statistic vs. constant model.

All data analysis was done in Matlab R2022a. All scripts and functions necessary to reproduce the results can be found in osf.io/kcd83.

## Results

We investigate if the short CART score is associated with: (1) measures extracted from the odd-one-out task, (2) the individual movement modalities recorded during the task and (3) the coordination patterns. All the statistical results, effect sizes and their significance levels can be found in Tables 3 and 4.

### Task Performance

**Figure 6.**
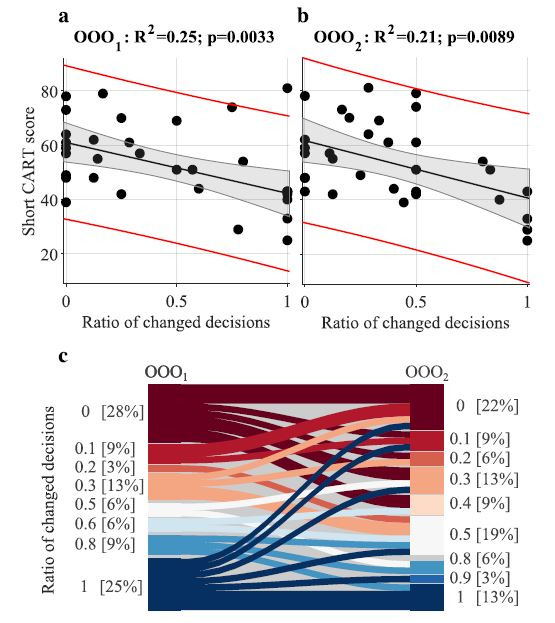
Correlation between ratio of the changed decisions in a) the 1^st^ round and b) the 2^nd^ round of the odd-one-out task and the short CART score. Black dots – indicate CART scores and corresponding ratios of changed decisions of individual participants, black line – fitted linear model, grey shaded region – 95% confidence bounds of the linear fit, red curves – 95% prediction bounds of the linear fit **c.** Sankey (flow) diagram illustrating change in distributions of the ratios of changed decisions in OOO_1_ and OOO_2_. Stacked bar plots show distribution of the ratios of changed decisions (rounded to a single decimal place). The connectors (flows) show change in behaviour of individual participants (they connect their ratios of changed decisions in OOO_1_ and OOO_2_).

We found that short CART score is correlated with ratios of changed decisions (see Table 4 and Figure 6) and with the mean time of the 1^st^ (initial) decisions (see Table 4). We further observe that in OOO_2_ only 4 participants always changed their decisions (8 in the OOO_1_) and 7 participants never changed their decision (9 in the OOO_1_). Time of initial decision was longer in the OOO_2_ (median_2_=8.2 sec.) than in OOO_1_ (median_1_=6.8 sec.); p=0.0077, Wilcoxon-Mann-Whitney test. Additionally, regression analysis shows that participants with higher CART score took more time to make the initial decision in OOO_2_.

### Movement Modalities (Velocity distributions)

Short CART score correlation with MDS coordinates show that the velocity distributions (and correlation matrices) are associated with the short CART scores (see Table 3). Correlations with lower MDS dimensions indicate that the association between short CART scores and head, hand and gaze velocities is stronger for the 2^nd^ OOO round. Analysis of associations of the velocity distributions and correlation matrices with the other task measures can be found in Supplementary Table 2.

**Table 3.** Statistical results for stepwise linear regression on MDS coordinates) with short CART score as a response variable.

	MDS coordinates	
	OOO_1_	OOO_2_
Head	**R^2^=0.18, p=0.017, (x_3_)**	**R^2^=0.19, p=0.013, (x_2_)**
Hand	**R^2^=0.25, p=0.0058, (x_6_)**	**R^2^=0.21, p=0.010, (x_1_)**
Gaze	**R^2^=0.24, p=0.015, (x_8_)**	**R^2^=0.16, p=0.042, (x_1_)**
Corr. Mat.	**R^2^=0.21, p=0.028, (x_2_)**	**R^2^=0.21, p=0.026, (x_2_)**

R^2^ – coefficient of determination of robust linear regression, p – p-value of F-statistic vs. constant model, (x_i_) – MDS coordinate with the strongest correlation (in terms of R^2^) found using the stepwise regression. Since the space defined by the MDS is abstract, directions of the association are irrelevant. In bold p-value < 0.05.

To interpret the significant correlations with the distribution from the 1^st^ dimension of the MDS in Table 3 we also analysed the association between the CART and the mean velocities of the actual movement modalities (see Table 4). The short CART scores were negatively correlated ( ρ < 0) with mean head velocity and mean hand velocity in the 2^nd^ round of the odd-one-out task.

**Table 4.** Statistical results for regression analysis with short CART score as a response variable.

	OOO_1_	OOO_2_
Ratio of changed decisions	𝛒**=-0.42,** **R^2^=0.25,** **p=0.0033**	𝛒 **=-0.48, R^2^=0.21, p=0.0089**
Mean time of 1st decision	ρ=0.29, R^2^=0.078, p=0.12	𝛒 **=0.36, R^2^=0.13, p=0.043**
Mean time of 2nd decision	ρ=0.12, R^2^=0.013, p=0.53	ρ=-0.17 R^2^=0.23, p=0.4
Total time	ρ=0.29, R^2^=0.094 p=0.087	ρ=0.16 R^2^=0.02, p=0.43
Mean head velocity	ρ=-0.061, R^2^=0.0033, p=0.76	𝛒 **=-0.36, R^2^=0.12, p=0.048**
Mean hand velocity	ρ=-0.11, R^2^=0.0085, p=0.63	𝛒**=-0.46** **R^2^=0.19, p=0.013**
Mean eye velocity	ρ=0.25, R^2^=0.059. p=0.25	𝛒**=0.42** **R^2^=0.16, p=0.044**
Saccade rate, #saccades/sec	ρ=0.29, R^2^=0.082. p=0.18	𝛒**=0.44** **R^2^=0.18, p=0.031**
Riemannian distance between coordination patterns in the two odd-one-out task rounds, RD(OOO_1_, OOO_2_)	𝛒**=-0.53,** **R^2^=0.25,** **p=0.018**		

Note. ρ – Pearson's linear correlation coefficient, R^2^ – coefficient of determination of robust linear regression, p – p-value of F-statistic vs. constant model for the robust regression. In bold p-value < 0.05.

**Figure 7.**
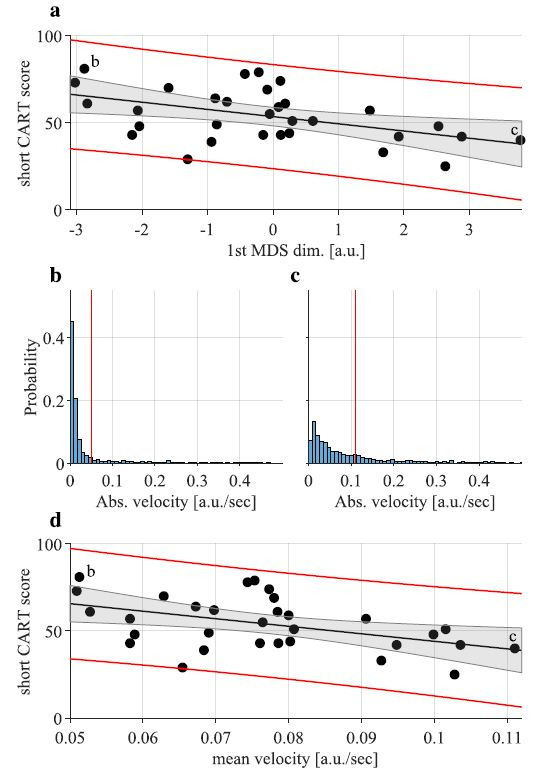
**a.** correlation between x-coordinate (1^st^ MDS dimension) of points representing distributions of absolute velocity of hand movements recorded in the OOO_2_ and the short CART score. Colours and symbols are the same as in Figure 6. **b.** and **c.** examples of the two distributions of absolute hand velocities indicate with b (short CART score 81) and c (short CART score 40) in panel a. red vertical line indicates mean velocity (b – 0.051[a.u./sec] and c – 0.11 [a.u./sec]) **d.** correlation between mean velocity of hand movements recorded in the OOO_2_ and the short CART score.

Figure 7 provides an illustration of the correlations between the short CART scores and the 1^st^ MDS dimension of the hand movement distributions (a – from Table 3) as well as with the mean hand movement velocity (d – from Table 4). It also shows two examples of the distributions of the absolute hand velocities (b and c). The figure shows that the 1^st^ MDS dimension of the abstract geometric space captures variability of the mean hand movement velocity. This is often, but not always, the case when analysing outcomes of the MDS of probability distributions (46). The head data patterns were very similar to those for hand movements and are not shown (mean head velocity is correlated with the 2^nd^ MDS dimension).

In contrast to the head and hand data, the short CART scores are *positively* correlated ρ > 0 with mean gaze velocity (Table 4). Participants with higher CART scores had faster eye movements. More specifically they have more saccades as confirmed by the positive correlation with saccade rate.

### Coordination Patterns

Analysis of the correlation matrices showed existence of an association between both OOO rounds and the short CART scores (Table 3). Since correlation matrix has 28 unique entries and can be parametrised in multiple ways (e.g., average correlation, maximum correlation, average correlation of head, hand gaze, etc.) the study is underpowered to precisely interpret correlations between which variables are driving the observed associations.

**Figure 8.**
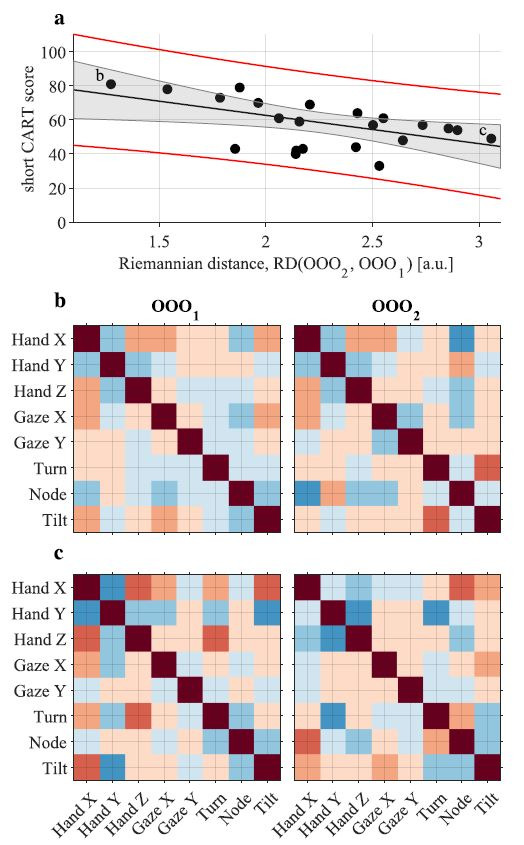
**a.** correlation of the Riemannian distance between correlation matrices from the two OOO rounds, RD(OOO_1_, OOO_2_), and the short CART score. Colours and symbols are the same as in Figure 6. **b.** shows two correlation matrices with of the participant that had the smallest change in the coordination pattern between the OOO_1_ (left) and OOO_2_ (right) **c.** shows two correlation matrices with of the participant that had the largest change in the coordination pattern between the OOO_1_ (left) and OOO_2_ (right).

## Discussion

In the presented work we sought insights into the ‘how’ and ‘why’ of individual, group and population behaviour, enabling predictions about how they are likely to act in the future. We explored ideas related to decision-making in uncertain conditions and particularly with respect to the effect of ambiguity (in the odd-one-out task particularly; see 42).

This exploratory work was novel in a number of ways, including (1) the use of AR to present tasks, (2) the development of novel experimental tasks to test rational thinking, (3) the assessment of physiological data related to how participants completed the task, and (4) the use of novel data analysis techniques. In summary, we demonstrated that it is possible to relate aspects of rational thinking with quantitative measures recorded in an interactive task taking place in augmented reality. As the CART – even in its short form – is a lengthy and somewhat abstract test, there is certainly potential for novel objective tasks to generate markers of important elements of rational thinking (5).

### Synthesis of findings

In the odd-one-out task, we found that more rational thinkers (higher short CART scores) were less likely to change their decisions when provided with this opportunity, and when presented with information that might challenge their initial response (see Table 4 and Fig. 6). In the more ambiguous OOO_2,_ participants presented fewer extreme behaviours than in the OOO_1_; fewer people never changed the initial decision and fewer people always changed the initial decision. They also took more time to make initial decision in the more ambiguous OOO_2_ round.

We also showed that our objective measures of motor behaviour (separate movement velocity distributions for eye, head and hand) and coordination patterns (eye-head-hand coordination) were associated with the overall short CART score (Tables 3 and 4). Specifically, we observed that participants with higher short CART scores moved their eyes more quickly but moved their head and hands more slowly than their less rational counterparts. This might reflect more deliberate and planned movements. They also maintained more similar eye-head-hand coordination patterns across both odd-one-out rounds, despite increased ambiguity (Table 4 and Fig. 8).

Overall that data collected in the 2^nd^ OOO round shows stronger associations with the short CART scores. This is probably unsurprising, as the 2^nd^ OOO round was more ambiguous (animals vs inanimate objects) and there was a higher chance that the additional information presented could include information that the participant did not consider when making their initial selection of the odd-one-out animal. Correlation of the short CART score with rate of saccades indicates that participants with higher CART scores might have different ways of analysing the displayed objects when choosing the odd-one-out element. Note that other eye-tracking measures (fixation rate, search rate and gaze transition entropy) were not correlated with the short CART scores (see supplementary Table 3).

While it is difficult to interpret these results in terms of specific task strategies, it suggests that more rational participants had a more coordinated process in gathering information and selecting options than their less rational counterparts; a process which helped them to be more confident in their initial choices. Previous research has shown that top-down attention drives our coordinated eye-head-hand behaviour in natural environments (1; 23). With experience, we learn to conserve limited cognitive resources and strategically direct our gaze control system to maximize information acquisition and guide accurate, goal-directed movement (23). A specific example that aligns with our current findings is an eye movement study by Jovancevic-Misic and Hayhoe (18). These authors showed that participants learn to attend to important events in the environment; with the time taken to first fixate on the stimulus decreasing for important events as participants become more experienced with the task.

Our findings reinforce the benefits of applying advanced statistical methods to the assessment of how systems coordinate (i.e., controlling eye, head, and hand movements) when trying to understand complex behaviour. Indeed, it has been suggested that it is important to consider how information provided by the entire body and its coordination dynamics, influences the way we make decisions (e.g., 34). Such embodied cognition – the view that cognitive dynamics are grounded in the way our body interacts with its physical and social environments – is arguably even more relevant to decision-making in tasks which involve consideration of what the body can do to enact decisions in the environment (see work in sport, 2). Our preliminary findings, suggest that it might be possible to establish novel mechanistic ways of understanding the complex relations between individual coordination strategies, behaviour and decision making in real-world environments where the quality of the movements themselves are important (e.g., sport, rehabilitation, defence and security, aviation, surgery etc.).

One critical issue to consider in real world, uncertain time-constrained environments, is the degree to which non-rational thinking is a problem and whether intuition might be useful, or even a characteristic of expertise (14; 22). For example, Klein and other naturalistic decision-making (NDM) researchers view intuition as an expression of experience, as people learn patterns that enable them to rapidly size up situations and make rapid decisions without having to compare options (see 22 for a recent discussion). In the real-world, the ‘mindware problems’ outlined in the CART (49) become more about the identification of task-specific patterns learned over time (and through training). As this research develops, the interplay between the NDM and ‘heuristic and biases’ fields will need more careful examination.

### Limitations

As a pilot study, this work is testing proof of principle, and as such our results should be interpreted with caution. There are dozens of separate biases referred to in the literature (e.g., 19) and we selected one that arose from our initial task planning work. It is perhaps not surprising that this distinct bias was only partially related to such a comprehensive measure as the CART. Additionally, it is possible that we are conflating susceptibility to biases to the use of an availability heuristic, or other personality traits such as openness to persuasion. For example, it is known that individuals might be persuaded to change choices based on additional (and recent) contextual information from an ‘expert’ (30). While it might have been useful to examine the relationships between specific factors of the CART and our objective measures, this was not allowed in the terms of the contract signed for publishing CART data (48). It is noteworthy that since we conducted this research, new measures for rational thinking are emerging which are more multi-dimensional (e.g., 5). Furthermore, we expect that the results should be replicable using the computer or mobile devices screens. Such replication would be very valuable.

### Future Research/ Exploitation

There are a number of future directions for this research to take. Currently, the analysis takes place offline as there are significant pre-processing and computational demands. It would be interesting to explore if we could get similar classification for online detection and feedback – something that will be important if we are to intervene at the point where thinking errors might be prevalent. Second, the exploration of the effect of different types of prompts (e.g., the modality by which they are presented, their linguistic form, their timing, etc.) will be important as this work moves into more ecologically valid settings. There is evidence that information presented via video is more readily believed than information presented in text format (52). Third, it would be interesting to explore how participants might be more or less biased by information presented in AR when compared to the real world. For example, the current odd-one-out task could be modified so that two items are presented in AR and two on a table, to see if there is consistent bias for one or the other. This might be relevant when it comes to operators making decisions based on AR information compared to the information they draw from their ‘own’ senses. A key heuristic related to both the modality of presentation and the use of AR is the realism heuristic, or the rule of thumb that “if something seems real, then it is credible” (51).

Fourth, task specific odd-one-out environments could be generated that provide more realistic scenarios and advice (or ‘case history’ contextual information). Such hypothetical clinical scenarios and vignettes are used regularly when assessing biases in medical decision making (6). The impact of such information is an important consideration for biases and decision making in a number of fields. For example, it is believed that up to 75% of errors in internal medicine practice are thought to be cognitive in origin, and errors in cognition have been identified in all steps of the diagnostic process, including information gathering, association triggering, context formulation, processing and verification (33). Similar issues with the provision of contextual information are evident in forensic science (30) and in policing, given the role of bias in use of force decisions (27).

To conclude, our study presents some promising results evidencing the potential pathways for developing objective measures of cognitive biases. It also clearly demonstrates advantages of going beyond gaze analysis in this area of research. The main benefits being potential insights into behavioural strategies and ability to compensate for lower quality of the eye-tracking data.

### Ethics and Conflict of Interest

The author(s) declare(s) that the contents of the article are in agreement with the ethics described in http://biblio.unibe.ch/portale/elibrary/BOP/jemr/ethics.html and that there is no conflict of interest regarding the publication of this paper.

### Acknowledgements

This research was supported in part by Phase 1, Defence and Security Accelerator (Behavioural Analytics) Contract Number DSTLX1000133918. Piotr Słowiński was generously supported by the Wellcome Trust Institutional Strategic Support Award 204909/Z/16/Z.

We wish to thank Jade Drain for help with setting up the Qualtrics version of the CART and assistance with data collection. We wish to thank Antonia Nash for assistance with data collection.

We would further like to acknowledge work of Julian Stopher on developing the AR tasks.

## supplementary material


