# Supplementary material for: Assessment of cognitive biases in Augmented Reality: Beyond eye tracking
Source: J Eye Mov Res. 2022 Dec 30;15(3):10.16910/jemr.15.3.4. doi: 10.16910/jemr.15.3.4 (PMC10171922; doi:10.16910/jemr.15.3.4)
Supplement: Supplementary file 1 [file jemr-15-03-d-SD1-01.pdf]

## Supplementary Note 1. Bias corrected distance correlation

Bias corrected distance correlation (BCDC) allowed us to directly analyse pairwise similarities/ distances between participants movements computed by means of earth's mover distance and Riemannian distance. To quantify and assess existence of associations we computed BCDC between all the pairwise similarities/ distances with all the pairwise differences between short CART scores of participants. BCDC measures all types of dependence between random variables in arbitrary, not necessarily equal dimensions. Distance correlation takes values in [0, 1] and is equal to zero if independence holds. It provides a scalar measure that characterizes independence of random vectors (Székely & Rizzo, 2013). In our analysis we are using the BCDC as an indicator of existence of an association. The p-value of BCDC is based on the student t-statistics of the BCDC correlation coefficient.

The low values of the BCDC ($\rho_{BC}$) signify large degree of additional variability captured by the markers. Significant BCDC indicate existence of a relation between short CART scores and head, hand and gaze velocities in the $OOO_2$.

Supplementary Table 1. Statistical results for distance-based analysis (BCDC) of associations of velocity distributions and correlations matrices with mean time of the 1st decision, mean time of the 2nd decision, ratio of changed decisions and total time as a response variable (column 1 the 1st round, column 2 the 2nd round).

| | $OOO_1$ | | | | | $OOO_2$ | | | | |
|---|---|---|---|---|---|---|---|---|---|---|
| | Short CART score | Mean time of 1st decision $OOO_1$ | Mean time of 2nd decision $OOO_1$ | Ratio of changed decisions $OOO_1$ | Total time $OOO_1$ | Short CART score | Mean time of 1st decision $OOO_2$ | Mean time of 2nd decision $OOO_2$ | Ratio of changed decisions $OOO_2$ | Total time $OOO_2$ |
| Head | $\rho_{BC}$=-0.027 p=0.71 T(433)=-0.55 | $\rho_{BC}$=0.073 p=0.064 T(433)=1.52 | $\rho_{BC}$=-0.036 p=0.77 T(433)=-0.74 | $\rho_{BC}$=-0.051 p=0.85 T(433)=-1.06 | $\rho_{BC}$=0.01 p=0.41 T(433)=0.22 | **$\rho_{BC}$=0.12 p=0.0059 T(463)=2.53** | $\rho_{BC}$=-0.021 p=0.67 T(463)=-0.45 | $\rho_{BC}$=-0.055 p=0.88 T(463)=-1.2 | $\rho_{BC}$=0.0084 p=0.43 T(463)=0.18 | $\rho_{BC}$=0.0098 p=0.42 T(463)=0.21 |
| Hand | $\rho_{BC}$=-0.043 p=0.80 T(376)=-0.83 | $\rho_{BC}$=0.033 p=0.26 T(376)=0.64 | $\rho_{BC}$=0.067 p=0.096 T(376)=1.3 | $\rho_{BC}$=-0.026 p=0.69 T(376)=-0.5 | $\rho_{BC}$=0.074 p=0.074 T(376)=1.4 | **$\rho_{BC}$=0.091 p=0.028 T(433)=1.91** | $\rho_{BC}$=0.018 p=0.35 T(433)=0.38 | $\rho_{BC}$=-0.052 p=0.86 T(433)=-1.1 | $\rho_{BC}$=0.057 p=0.12 T(433)=1.2 | $\rho_{BC}$=0.002 p=0.48 T(433)=0.042 |
| Gaze | $\rho_{BC}$=-0.07 p=0.87 T(251)=-1.11 | $\rho_{BC}$=0.0014 p=0.49 T(251)=-0.023 | $\rho_{BC}$=0.036 p=0.29 T(251)=0.57 | $\rho_{BC}$=0.063 p=0.16 T(251)=1 | $\rho_{BC}$=0.015 p=0.4 T(251)=0.25 | **$\rho_{BC}$=0.097 p=0.046 T(298)=1.69** | $\rho_{BC}$=-0.067 p=0.88 T(298)=-1.17 | $\rho_{BC}$=-0.0037 p=0.53 T(298)=-0.06 | $\rho_{BC}$=-0.065 p=0.87 T(298)=-1.12 | $\rho_{BC}$=-0.011 p=0.57 T(298)=-0.18 |
| Corr. Mat. | **$\rho_{BC}$=0.21 p=0.00038 T(251)=3.41** | $\rho_{BC}$=0.044 p=0.24 T(251)=0.69 | $\rho_{BC}$=-0.031 p=0.69 T(251)=-0.49 | $\rho_{BC}$=-0.036 p=0.71 T(251)=-0.57 | $\rho_{BC}$=0.064 p=0.16 T(251)=1.01 | **$\rho_{BC}$=0.10 p=0.042 T(274)=1.74** | $\rho_{BC}$=0.099 p=0.051 T(274)=1.64 | $\rho_{BC}$=0.03 p=0.31 T(274)=0.5 | $\rho_{BC}$=-0.018 p=0.62 T(274)=0.3 | $\rho_{BC}$=0.027 p=0.33 T(274)=0.45 |

Note. $\rho_{BC}$ – bias corrected Pearson's linear correlation coefficient, p – p-value of the student t statistics, T(DF) - the student t statistics and its degree of freedom DF. Since the space defined by the MDS is abstract, directions of the association are irrelevant. In bold p-value < 0.05.

Supplementary Table 2. Statistical results for distance-based analysis (stepwise linear regression on MDS coordinates) of associations of velocity distributions and correlations matrices with mean time of 1st decision, mean time of 2nd decision, ratio of changed decisions and total time as a response variable (column 1 the 1st round, column 2 the 2nd round).

| | $OOO_1$ | | | | $OOO_2$ | | | |
|---|---|---|---|---|---|---|---|---|
| | Mean time of 1st decision $OOO_1$ | Mean time of 2nd decision $OOO_1$ | Ratio of changed decisions $OOO_1$ | Total time $OOO_1$ | Mean time of 1st decision $OOO_2$ | Mean time of 2nd decision $OOO_2$ | Ratio of changed decisions $OOO_2$ | Total time $OOO_2$ |
| Head | **$R^2$=0.37 p=0.0003 ($x_2$)** | **$R^2$=0.14 p=0.036 ($x_3$)** | $R^2$=0.015 p=0.51 ($x_1$) | **$R^2$=0.19 p=0.013 ($x_3$)** | $R^2$=0.02 p=0.44 ($x_1$) | $R^2$=0.0083 p=0.62 ($x_1$) | **$R^2$=0.17 p=0.019 ($x_2$)** | $R^2$=0.043 p=0.25 ($x_1$) |
| Hand | $R^2$=0.1 p=0.091 ($x_1$) | $R^2$=0.1 p=0.087 ($x_1$) | $R^2$=0.018 p=0.49 ($x_1$) | $R^2$=0.11 p=0.076 ($x_1$) | $R^2$=0.063 p=0.17 ($x_1$) | $R^2$=0.016 p=0.5 ($x_1$) | **$R^2$=0.14 p=0.04 ($x_1$)** | $R^2$=0.0068 p=0.66 ($x_1$) |
| Gaze | **$R^2$=0.3 p=0.0054 ($x_2$)** | $R^2$=0.0043 p=0.76 ($x_1$) | $R^2$=0.16 p=0.056 ($x_1$) | **$R^2$=0.28 p=0.0084 ($x_2$)** | $R^2$=0.023 p=0.46 ($x_1$) | $R^2$=0.07 p=0.19 ($x_1$) | $R^2$=0.041 p=0.32 ($x_1$) | $R^2$=0.025 p=0.44 ($x_1$) |
| Corr. Mat. | $R^2$=0.081 p=0.68 ($x_1$) | $R^2$=0.0020 p=0.84 ($x_1$) | $R^2$=0.0038 p=0.78 ($x_1$) | $R^2$=0.006 p=0.73 ($x_2$) | $R^2$=0.076 p=0.19 ($x_1$) | **$R^2$=0.33 p=0.0034 ($x_1$)** | $R^2$=0.013 p=0.59 ($x_1$) | $R^2$=0.027 p=0.44 ($x_1$) |

Note. $R^2$ – coefficient of determination of robust linear regression, p – p-value of F-statistic vs. constant model, ($x_i$) – MDS coordinate with the strongest correlation (in terms of $R^2$) found using the stepwise regression. Since the space defined by the MDS is abstract, directions of the association are irrelevant. In bold p-value < 0.05.

Supplementary Table 3. Statistical results for regression analysis with short CART score as a response variable.

| | $OOO_1$ | $OOO_2$ |
|---|---|---|
| **Fixation rate**<br>Number of visual fixations (periods in which the eye was stable on a location) made per second. Fixations were defined as clusters of successive gaze point records that fell within 0.03 of the normalized world frame for >100ms (Salvucci &Goldberg, 2000). | $\rho$=0.3, $R^2$=0.083, p=0.17 | $\rho$=0.17, $R^2$=0.021, p=0.48 |
| **Search rate**<br>Number of fixations divided by their total duration. | $\rho$=0.31, $R^2$=0.091, p=0.15 | $\rho$=0.39, $R^2$=0.15, p=0.055 |
| **Gaze transition entropy (GTE)**<br>Gaze transition entropy quantifies the complexity of the gaze trajectory in 'bits', (Lounis et al., 2021). GTE is defined as:<br>$GTE = -\sum_{i=1}^{n} p(i) \sum_{j=1}^{n} p(j\|i) \log_2 p(j\|i)$<br>With $p(i)$ being the probability of being in a region *i* and $p(j\|i)$ being the conditional probability of moving from region *i* to *j*. To define the regions, we divided the world frame into 100 equal squares (10 by 10 grid). | $\rho$=0.14, $R^2$=0.018, p=0.53 | $\rho$=-0.29 $R^2$=0.012, p=0.09 |

Note. $\rho$ – Pearson's linear correlation coefficient, $R^2$ – coefficient of determination of robust linear regression, p – p-value of F-statistic vs. constant model for the robust regression. In bold p-value < 0.05.

Supplementary Table 4. Statistical results for regression analysis with mean time of 1st decision, mean time of 2nd decision, ratio of changed decisions and total time as a response variable (column 1 the 1st round, column 2 the 2nd round).

| | $OOO_1$ | | | | $OOO_2$ | | | |
|---|---|---|---|---|---|---|---|---|
| | Mean time of 1st decision $OOO_1$ | Mean time of 2nd decision $OOO_1$ | Ratio of changed decisions $OOO_1$ | Total time $OOO_1$ | Mean time of 1st decision $OOO_2$ | Mean time of 2nd decision $OOO_2$ | Ratio of changed decisions $OOO_2$ | Total time $OOO_2$ |
| Sacc rate | $\rho$=0.22 $R^2$=0.061 p=0.24 | $\rho$=0.072 $R^2$=0.0068 p=0.7 | $\rho$=0.39 $R^2$=0.14 p=0.071 | $\rho$=0.16 $R^2$=0.026 p=0.45 | $\rho$=-0.021 $R^2$=0.019 p=0.85 | $\rho$=-0.32 $R^2$=0.065 p=0.24 | $\rho$=-0.19 $R^2$=0.033 p=0.41 | $\rho$=-0.25 $R^2$=0.053 p=0.26 |
| Fix Rate | **$\rho$=0.37 $R^2$=0.22 p=0.022** | $\rho$=0.27 $R^2$=0.089 p=0.16 | **$\rho$=0.42 $R^2$=0.17 p=0.045** | **$\rho$=0.34 $R^2$=0.18 p=0.038** | $\rho$=0.038 $R^2$=0.022 p=0.77 | $\rho$=0.028 $R^2$=0.017 p=0.8 | $\rho$=-0.088 $R^2$=0.0085 p=0.76 | $\rho$=0.053 $R^2$=0.01 p=0.69 |
| Search rate | $\rho$=0.29 $R^2$=0.13 p=0.082 | $\rho$=0.13 $R^2$=0.023 p=0.48 | $\rho$=0.36 $R^2$=0.14 p=0.076 | $\rho$=0.24 $R^2$=0.087 p=0.16 | $\rho$=0.028 $R^2$=0.022 p=0.97 | $\rho$=-0.35 $R^2$=0.085 p=0.16 | $\rho$=-0.23 $R^2$=0.049 p=0.29 | $\rho$=-0.22 $R^2$=0.036 p=0.36 |
| GTE | **$\rho$=0.32 $R^2$=0.34 p=0.0029** | **$\rho$=0.3 $R^2$=0.27 p=0.0086** | $\rho$=0.19 $R^2$=0.032 p=0.4 | **$\rho$=0.39 $R^2$=0.5 p=0.0001** | **$\rho$=0.21 $R^2$=0.42 p=0.003** | **$\rho$=0.47 $R^2$=0.16 p=0.046** | $\rho$=0.069 $R^2$=0.008 p=0.74 | **$\rho$=0.48 $R^2$=0.27 p=0.0073** |

Note. $\rho$ – Pearson's linear correlation coefficient, $R^2$ – coefficient of determination of robust linear regression, p – p-value of F-statistic vs. constant model for the robust regression. In bold p-value < 0.05. Correlations of GTE with time variables are expected since longer signal has a higher chance of being complex.

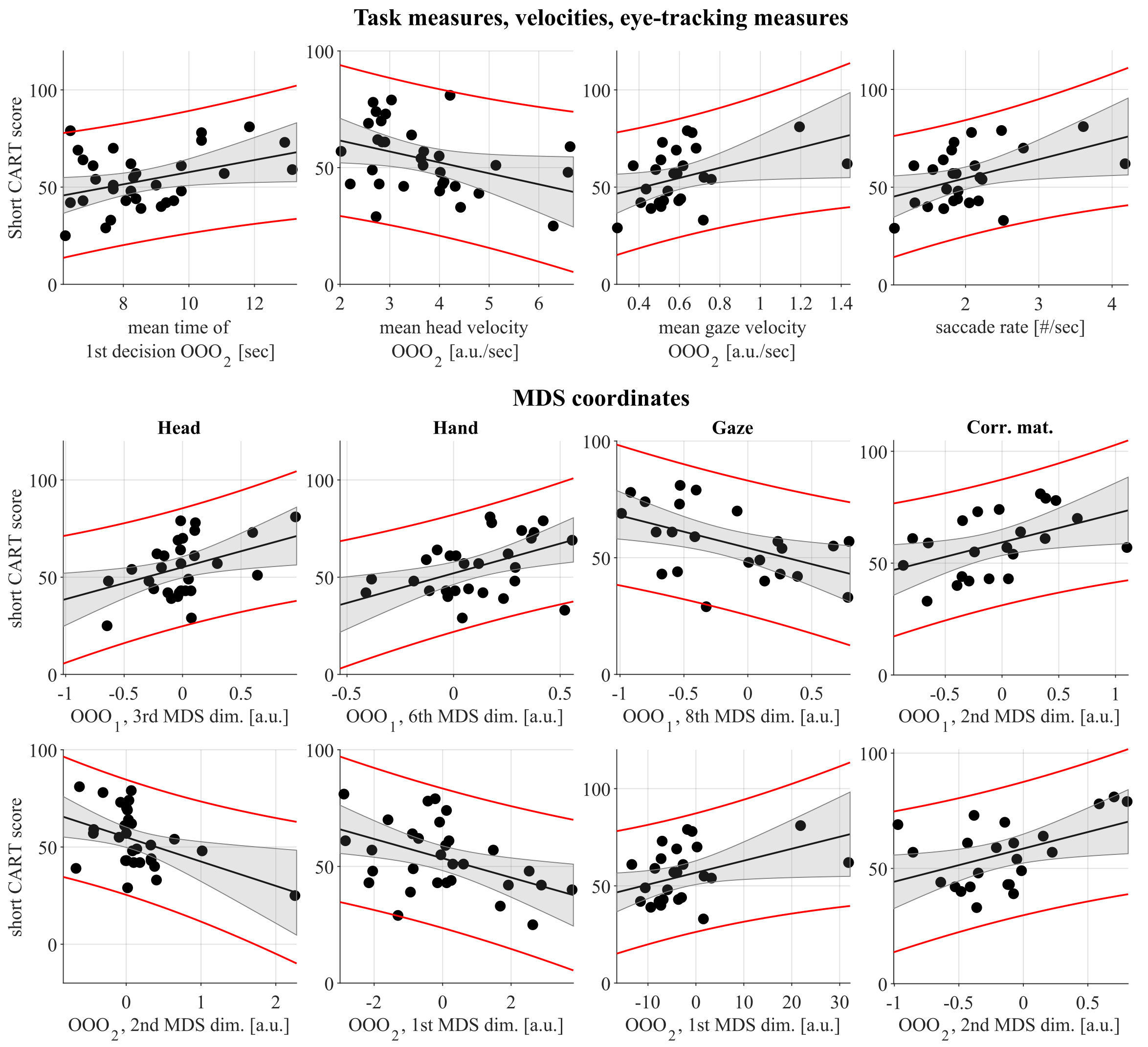


Supplementary Figure 1. Scatter plots illustrating correlations of task measures, mean velocities, eye-tracking measures and MDS coordinates with the short CART score reported in Tables 3 and 4 (not shown in previous figures, except the 2nd panel in the bottom row, Hand $OOO_2$, which is the same as Fig. 7a).
